# Production and Characterization of Keratinolytic Protease from New Wool-Degrading *Bacillus* Species Isolated from Egyptian Ecosystem

**DOI:** 10.1155/2013/175012

**Published:** 2013-07-11

**Authors:** Mohamed A. Hassan, Bakry M. Haroun, Amro A. Amara, Ehab A. Serour

**Affiliations:** ^1^Protein Research Department, Genetic Engineering and Biotechnology Research Institute, City of Scientific Research and Technological Applications, New Borg Al-Arab, P.O. Box. 21934, Alexandria, Egypt; ^2^Botany and Microbiology Department, Faculty of Science (Boys), Al-Azhar University, Cairo, Egypt; ^3^King Abdulaziz City for Science and Technology, Riyadh, Saudi Arabia

## Abstract

Novel keratin-degrading bacteria were isolated from sand soil samples collected from Minia Governorate, Egypt. In this study, the isolates were identified as *Bacillus amyloliquefaciens *MA20 and *Bacillus subtilis *MA21 based on morphological and biochemical characteristics as well as 16S rRNA gene sequencing. *B. amyloliquefaciens *MA20 and *B. subtilis *MA21 produced alkaline keratinolytic serine protease when cultivated in mineral medium containing 1% of wool straight off sheep as sole carbon and nitrogen source. The two strains were observed to degrade wool completely to powder at pH 7 and 37°C within 5 days. Under these conditions the maximum activity of proteases produced by *B. amyloliquefaciens *MA20 and *B. subtilis *MA21 was 922 and 814 U/ml, respectively. The proteases exhibited optimum temperature and pH at 60°C and 9, respectively. However, the keratinolytic proteases were stable in broad range of temperature and pH values towards casein Hammerstein. Furthermore the protease inhibitor studies indicated that the produced proteases belong to serine protease because of their sensitivity to PMSF while they were inhibited partially in presence of EDTA. The two proteases are stable in most of the used organic solvents and enhanced by metals suggesting their potential use in biotechnological applications such as wool industry.

## 1. Introduction

Keratins are classified as fibrous proteins called scleroproteins that occur abundantly in epithelial cells. These proteins are insoluble in water, weak acid and alkali, and organic solvents and are insensitive to the attack of common proteolytic enzymes such as trypsin or pepsin [1]. The animal remains rich in **α**-keratin such as animal skin, hair, claws, horns, and wools.

The important property of these proteins is the presence of high cystine content that differentiates keratins from other structural proteins such as collagen and elastin. Both a high cystine content as well as a high content of glycine, proline, serine, and acidic amino acids and a low content of lysine, histidine, and methionine (or their lack) as well as the absence of tryptophan are also characteristic of keratins [2, 3]. Numerous disulfide cystine bonds present in keratin to bind peptide chains and packed as **α**-helices as in hair and wool or in **β**-sheet arrangements as in case of feathers. The disulfide linkage and the tight secondary structure of keratins make them difficult to be hydrolysed by common proteolytic enzymes [4].

The major problem of *α*-keratin hydrolysis is the presence a high numbers of disulfide bonds that make it insoluble in nature and resistant to proteases hydrolysis [5]. Keratinolytic protease enzymes are spread in nature and elaborated by different groups of microorganisms that can be isolated from polluted area with keratin wastes [6]. A vast variety of Gram-positive bacteria including *Bacillus*, *Lysobacter*, *Nesternokia*, *Kocuria,* and *Microbacterium* as well as a few strains of Gram-negative bacteria such as *Xanthomonas*, *Stenotrophomonas,* and *Chryseobacterium* are confined as keratin degraders [7–10]. Most of keratin degrading bacteria belong to the genus of *Bacillus* [11].

The keratinous substrate such as feather and wool can be degraded in basal medium by microorganisms which are capable of utilize keratin as sole carbon and nitrogen source [12]. Keratinolytic proteases have broad substrate specificity where they have the ability to hydrolyze soluble protein such as casein, gelatin, and bovine serum albumin. Additionally, they can hydrolyze the insoluble protein including feather, silk, and wool [13]. Keratinolytic proteases mostly belong to serine or metalloproteases showing sequence similarity with subtilisin group of proteases [14, 15]. In recent years, more demands to keratinolytic proteases are increasing due to their multitude in industrial applications such as the feed, fertilizer, detergent, and textile industries. The present study describes the isolation and identification of new *Bacillus amyloliquefaciens* MA20 and *B. subtilis* MA21 strains from Egyptian ecosystem that grow well on wool as sole carbon source. Moreover the two strains are able to degrade wool and their enzymes can be used to improve the wool quality. This paper includes full characterization of the keratinolytic protease which explains that the best environmental conditions can be used to improve the wool quality in industry.

## 2. Materials and Methods

### 2.1. Sample Collection

Different types of samples were collected from different Governorates of Egyptian ecosystem included Alexandria, Behera, Qaliubia, Mania, and Asiut. These fresh samples were varied such as soil, sand soil, humus, waste wool, and rhizosphere under olive trees. These samples were collected in sterile falcon tubes and transported to the microbiological lab in (City of Scientific Research and Technological Applications).

### 2.2. Strains Isolation

The bacterial strains were isolated by suspending 1 g of soil samples in a 10 mL sterile 0.85% (w/v) saline solution and then treated for 20 min at 80°C. This will enable the isolation of the spore-forming bacteria. Luria-Bertani (LB) agar medium with 1% (w/v) skim milk was used for their cultivation by spreading 0.1 mL of each 10^−5^ and 10^−6^ dilutions. The plates then were incubated for 24 hours at different temperatures [4, 16]. The colonies which give clear zones formed by hydrolysis of skim milk were picked. Pure bacterial isolates were obtained by reculturing individual colonies several times on fresh LB agar medium to produce single colony from each.

### 2.3. Strains Selection

Twelve selected strains isolated according to the diameter of clear zone were cultured on medium containing 0.5 g NaCl, 0.3 g K_2_HPO_4_, 0.4 g KH_2_PO_4_, and 10 g wool per liter; pH 7 and incubated for 5 days at 37°C. The wool was used as sole carbon and nitrogen source for detecting potent strains that have the ability to degrade the wool completely. Three strains degraded the wool and the supernatants of their culture were assayed on plate containing 1% gelatin powder which is soluble in phosphate buffer pH 7. After determining the existence of the activity (by the clear zone of the supernatants), *Bacillus *sp. MA20 and *Bacillus *sp. MA21 were selected and preserved for further investigation.

### 2.4. Bacterial Identification

While the phenotypic characteristics and isolation method of the two selected isolates indicate that they are related to *Bacillus* group but further identification was conducted. 

The strains identification are included the spore morphology, Gram stain, and motility. The morphological and physiological characteristics of the bacterial isolates were compared with the data from Bergey's Manual of Determinative Bacteriology [17].

### 2.5. Scanning of *Bacillus *sp. MA20 and *Bacillus *sp. MA21 by Scanning Electron Microscope

The bacterial smear was prepared by centrifuging the bacterial cultures at 12,000 rpm for 20 min. The pellets were washed 2 times by saline solution. The pellet was suspended in sterilized distilled H_2_O. The bacterial film was prepared and fixed on glass slides till complete drying. The smear was coated with gold using sputter coater. The golden coated sample was scanned at 20 KV acceleration voltages at room temperature.

### 2.6. Genetic Identification and Differentiation

#### 2.6.1. DNA Extraction

The genomic DNA of *Bacillus* strains was isolated using modification method from Sambrook et al. [18].

#### 2.6.2. Identification by 16S Ribosomal RNA (rRNA)

*PCR Amplification according to Sambrook et al. [18]*. The PCR amplification reactions were performed in a total volume of 50 *μ*L. Each reaction mixture contained the following solutions: 2 *μ*L of DNA (40 ng), 1 *μ*L of 10 pmol forwarded 16S-rRNA primer (5′-AAATGGAGGAAGGTGGGGAT-3′); 1 *μ*L of 10 pmol reverse 16S rRNA primer (5′-AGGAGGTGATCCAACCGCA-3′); 0.8 *μ*L of 12.5 mM (dNTP's); 5 *μ*L of PCR buffer included MgCl_2_, and 0.2 *μ*L Taq polymerase (1 Unit) and water-free DNAse and RNAse were added up to 50 *μ*L. The PCR apparatus was programmed as follows: 3 min denaturation at 95°C, followed by 35 cycles that consisted of 1 min at 95°C, 1 min at 58°C, and 1 min at 72°C, and a final extension was 10 min at 72°C. The products of the PCR amplification were analyzed by agarose gel electrophoresis (2%).

#### 2.6.3. PCR Cleanup and 16S rRNA Sequencing

The PCR products were cleaned up for DNA sequencing following the method described by Sambrook et al. [18]. Automated DNA sequencing based on enzymatic chain terminator technique, developed by Sanger et al. [19], was carried out using 3130X DNA Sequencer (Genetic Analyzer, Applied Biosystems, Hitachi, Japan).

#### 2.6.4. Phylogenetic Analysis

Similarity analysis of the nucleotides was performed by BLAST searches against sequences available in GenBank. For phylogenetic tree construction, multiple sequences were obtained from GenBank and the alignments were performed using MEGA 5 software version 5.1 [20].

### 2.7. Keratinolytic Protease Production

*Bacillus *sp. MA20 and *Bacillus *sp. MA21 were first inoculated in liquid LB medium to produce large amount of cells. After 18 hrs, the colony forming units (CFU)/mL culture were 3∗10^6^, and 2% volume of the liquid medium was transferred to the production medium using 250 mL flask containing 100 mL of the production medium. The production medium containing (w/v) NaCl, 0.5 g/L; K_2_HPO_4_, 0.3 g/L; KH_2_PO_4_, 0.4 g/L; wool, 10 g/L; and the pH was adjusted 7.0–7.2 using 2N of NaOH and HCl [16]. The cultivated media were incubated at 37°C and 200 rpm for 5 days. Culture supernatants were obtained by centrifugation at 12,000 rpm and 4°C for 30 min. The different supernatants which contain the crude enzymes were used in assay and analysis of enzymes.

### 2.8. Enzymes Assay

#### 2.8.1. Detection of the Proteolytic Activity on Plates

The crude enzyme of *Bacillus *sp. MA20 and *Bacillus *sp. MA21 was screened for their proteolytic activity using agar well diffusion plate method described by Amara et al., after modification [21]. One gram of gelatin powder was suspended in 100 mL phosphate buffer pH 7 and autoclaved. After sterilization, the soluble component was added to sterile water containing agar (18 gm agar/L). The suspension then stirred gently and distributed in Petri dishes (25 mL/plate). After complete solidification of the agar on plates, wells were punched out of the agar, by using a clean sterile cork borer. The base of each hole was sealed with a drop of melted sterile water agar (15 g agar per liter H_2_O) using sterile Pasteur pipette. Fifty *μ*L of each bacterial supernatant was added to each well and preincubated at 4°C for 2 hrs and then overnight incubated at different temperatures.

#### 2.8.2. Visualization of the Enzyme Clear Zone

Coomassie blue (0.25%, w/v) in methanol-acetic acid-water 5 : 1 : 4 (v/v/v) was used in plates staining to visualize the gelatin hydrolysis where 10 mL was added to each plate and incubated in room temperature for 15 min followed by removing the staining solution from the plates surfaces and washing gently by distilled water. Then the plates were destained using destining solution (66 mL methanol, 20 mL acetic acid, and 114 mL H_2_O_bidest_) for a suitable time [22]. Also extracellular protease detection was determined according to Vermelho et al., [23] with modification. Staining was performed with 0.1% amido black in methanol-acetic acid-water 30 : 10: 60 (v/v/v) for 5–20 min (according to the quality of amido black stain) at room temperature. Regions of enzyme activity were detected as clear areas, indicating that hydrolysis of the substrates has been occurred.

#### 2.8.3. Enzyme Assay Spectrophotometrically

*Preparation of Casein in Different Buffers with Different pH*. Casein Hammarstein was weighted in a quantity of 0.325 g and dissolved in 50 mL of different buffers at different pH. The mixture was dissolved by heating gently to 80–90°C without boiling in water bath according to Amara and Serour [24] or using hot plate with stirring for more accurate because casein is highly stick with the walls of the container. The following buffers were used:

- sodium phosphate, pH 6-7-8 (0.1 M)
- glycine/NaOH pH 9-10 (0.1 M)
- sodium phosphate dibasic/NaOH pH 11 (0.05 M)
- KCl/NaOH pH 12 (0.2 M).

#### 2.8.4. Optimum Temperature of the Enzymes

Ten *μ*L of each supernatant which contains the crude enzyme was added to 490 *μ*L of the casein Hammarsten soluble in buffer pH 7. The enzymes-substrate mixture was incubated at different temperatures of 20, 30, 40, 50, 60, 70, and 80°C using water bath for 15 min. After the incubation period the enzyme reaction stopped by adding 500 *μ*L of 10% trichloroacetic acid (TCA). The mixture was allowed to stand in ice for 10 min and then centrifuged at 13,000 rpm for 10 min. The absorbance of each sample was determined spectrophotometrically at 280 nm and their tyrosine content derived from the tyrosine standard curve which was carried out according to Amara and Serour [24] and the enzyme activity was determined as Unit/mL.

#### 2.8.5. Optimum pH of the Enzymes

Ten *μ*L of each supernatant which contains the crude enzyme was added to the 490 *μ*L of the casein Hammarsten soluble in different pH values. The enzymes-substrate mixture was incubated at 60°C which acts as optimum temperature in a water bath for 15 min. At the end of incubation period the enzyme reaction stopped by adding 500 *μ*L of 10% trichloroacetic acid (TCA). The mixture was allowed to stand in ice for 10 min and then centrifuged at 13,000 rpm for 10 min. The absorbance of each sample was determined spectrophotometrically at 280 nm and their tyrosine content derived from the tyrosine standard curve and the enzyme activity determined as Unit/mL.

#### 2.8.6. Confirmation of Optimum pH and Temperature

The previous reaction was performed using casein Hammarsten soluble in Glycine/NaOH pH 9. The enzyme substrate mixture was incubated at various temperature of 20, 30, 40, 50, 60, 70 and 80°C in water bath for 15 min. The enzyme activity was calculated as mentioned previously.

#### 2.8.7. The Optimized Enzymes Reaction

The optimized reaction is a mixing between 10 uL of crude enzyme and 490 *μ*L of the casein Hammarsten soluble in Glycine/NaOH pH 9. The mixture was incubated at 60°C in water bath for 15 min. The reaction was stopped with 500 *μ*L of 10% TCA. The mixture was allowed to stand in ice for 10 min and then was centrifuged at 13,000 rpm for 10 min. The enzyme activity was determined as mentioned above using tyrosine standard curve.

#### 2.8.8. Thermal Stability

The thermostability was carried out by preincubating the crude enzyme solution at a temperature range of 4°C to 80°C for 0.0 to 24 hrs. The residual activity was measured with standard enzyme reaction. The control is enzyme reacted at zero time (consider as 100%) [16].

#### 2.8.9. pH Stability

The pH stability was determined by preincubating the enzyme solution in buffers with different pH values (3–12) at room temperature from 0.0 to 48 hrs. The residual activity was measured with standard enzyme reaction. The control is enzyme reacted at zero time and considered as 100% [16]. The following buffers were used:

- citrate buffer pH 3-4-5-6 (0.1 M)
- sodium phosphate pH 7-8 (0.1 M)
- glycine/NaOH pH 9-10 (0.1 M)
- sodium phosphate dibasic/NaOH pH 11 (0.05 M)
- KCl/NaOH pH 12 (0.2 M).

#### 2.8.10. Effect of Inhibitors

The inhibitors were added to supernatants which contain the produced enzyme and were incubated for 30 min at 30°C before being tested for proteolytic activity. Protease inhibitors phenylmethanesulphonyl fluoride (PMSF), ethylenediaminetetraacetic acid (EDTA), **β**-mercaptoethanol, and the detergent sodium dodecyl sulfate (SDS) were used. The inhibitors stocks were prepared in distilled water except that PMSF was prepared by using isopropanol. The final concentrations of PMSF, EDTA, and **β**-Mercaptoethanol are 5 mM and 1 mM while SDS is 0.5% and 0.1% (w/v). The control was enzyme mixed with distilled water instead of inhibitors. Control activity was considered to be 100% [25].

#### 2.8.11. Effect of Metal Ions

The effect of metal ions on protease activity was investigated using two concentrations of 5 mM and 10 mM (final concentration). The metal stock solutions were prepared in distilled water and diluted to the appropriate concentrations. The enzyme solution was mixed with the different metal solutions and incubated for 30 min at 30°C before assay. A control was also included where the enzyme was mixed with distilled water instead of metal solution. Control activity was considered to be 100% [25]. ZnCl_2_, MgCl_2_, CuSO_4_, Urea, HgCl_2_, CaCl_2_, BaCl_2_, Guanidin HCl, and MnCl_2_ were used.

#### 2.8.12. Effect of Solvents

The effect of the different solvents (Methanol, Ethanol, DMSO, Isopropanol, Tween 20, and Triton X100) on protease activity was investigated using a concentration of 1% and 0.5% (final concentration) [25].

### 2.9. First-Dimension Protein Electrophoresis

#### 2.9.1. Sodium Dodecyl Sulfate Polyacrylamid Gel Electrophoresis (SDS-PAGE)

Characterization of proteins and evaluation of the protein enrichment process SDS-PAGE was performed in a discontinuous SDS-PAGE vertical slab gel electrophoresis apparatus as described by Laemmli [26]. Discontinuous SDS-PAGE consisted of a stacking gel (5%, w/v, pH 6.8) and a separating gel (12%, w/v, pH 8.8). The separating gel was prepared in a 1 mm slab gel (10 × 10 cm).

### 2.10. Gel Staining Using Silver Stain

The gels were stained using silver nitrate staining methods as described by Blum et al. [27].

### 2.11. Zymogram for the Detection of Protease Activity Using SDS-PAGE

As above in the case of protease zymography using SDS PAGE and the separating gel concentration was 12%. The samples were mixed with 5X sample buffer without **β**-mercaptoethanol and without boiling. 

After running the gel at 80 V the SDS-PAGE gel was stripped off from the gel plate and soaked in 1% triton X100 for 2 hrs to change the solution every 1 hour or in 2.5% triton X100 for 30 min for removing SDS and renaturating the enzymes.

The gel was washed three times with tape water and soaked in 1% gelatin powder solubilized in Glycine/NaOH buffer pH 9 for 90 min at 60°C. The gel was washed with buffer for 10 min before staining. 

Then the gel was stained with Coomassie blue stain for 2 hrs. After that the gel was destained till the active bands appear.

### 2.12. Zymogram for Detecting Protease Activity Using Native PAGE

The gel was carried out using all components of the SDS-PAGE and the same conditions but without SDS as well as the buffers described above. The samples were prepared by mixing with 5X sample buffer for native PAGE and without boiling. The gel was washed with tap water and soaked into 1% gelatin powder as mentioned previously. Then the gel was stained with Coomassie blue stain for 2 hrs. After that, the gel was destained till the active bands appear.

### 2.13. Two-Dimension Polyacrylamide Gel Electrophoresis (2D-PAGE)

First-dimension isoelectric focusing (IEF) was performed using Ettan IPGphor3 and the second-dimension SDS-PAGE was applied using vertical electrophoresis of Ettan DALTtwelve System. The experiment was carried out using the operation instruction of manufacturer (GE healthcare *company*). The gels were stained with Coomassie stain where Coomassie blue R-250 (0.02 g) in methanol-glacial acetic acid-distilled water (10 mL, 5 mL, and 100 mL resp.) was used.

## 3. Results and Discussion

### 3.1. Strain Isolation

Microbial keratinolytic protease has been described for various biotechnological applications in food, detergent, textiles, and leather industries, and yet the growing demand for these enzymes necessitates the screening for novel keratinolytic microorganisms with potential applications [28, 29]. Keratinolytic protease has been described for several species of *Bacillus* [30, 31] due to the broad distribution of keratinase among these genera, and this study focused on keratinolytic protease production from them. 

**Figure 1:**
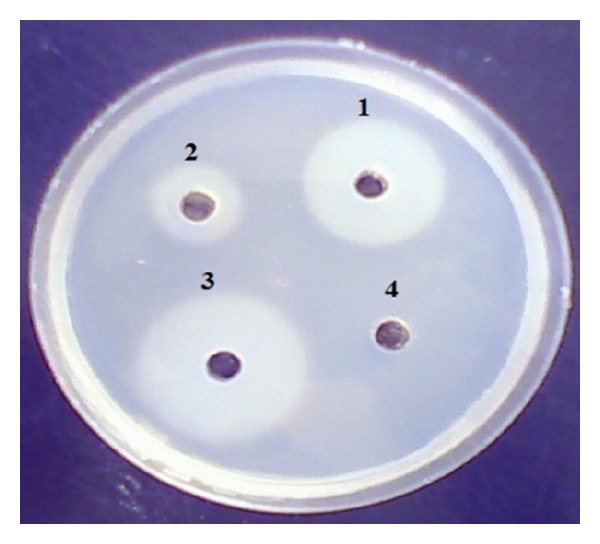
Proteolytic activity of the supernatants obtained from three *Bacillus *sp. on gelatin suspended in phosphate buffer pH 7. The well number (1) is supernatant from *Bacillus *sp. MA21, number (2) is from* Bacillus *sp. MA10, number (3) is from *Bacillus *sp. MA20, and (4) is control free of enzyme.

A total of 48 pure cultures of spore-forming bacteria were isolated and purified which obtained from different samples collected from Governorates of Egypt. All isolates were screened using selective method for *Bacillus* isolation. The proteolysis activities of all the isolates were detected using the plate test method containing LB agar medium with 1% (w/v) skim milk. Among the isolates analyzed, 12 isolates exhibited proteolytic activity in which they had a halo diameter of fivefold longer than the colony diameter. All isolates have the proteolytic activity but do not have the ability to degrade wool, for that the twelve selected isolates were grown using a medium which contain (w/v) NaCl, 0.5 g/L; K_2_HPO_4_, 0.3 g/L; KH_2_PO_4_, 0.4 g/L; wool, 10 g/L; and the pH was adjusted at 7.0–7.2 using 2N NaOH and HCl. The flasks were incubated for 5 days until the wool has been completely degraded by some isolates. Three *Bacillus* isolates show the ability to degrade the wool and gained the names *Bacillus *sp. MA20, *Bacillus *sp. MA21, and *Bacillus *sp. MA10. In the last screening step, the obtained supernatants from the cultivation of three above *Bacillus* strains culture media were assayed using agar well diffusion in Petri dishes including gelatin powder plate which suspended in phosphate buffer pH 7 as in Figure 1. *Bacillus *sp. MA20 and *Bacillus *sp. MA21 were selected for studying the keratinolytic protease enzyme based on the diameter of clear zone (Figure 1). By refering to their isolation source, the two selected strains were found to be isolated from Minia Governorates. This method is simple but proves to be efficient for determining the best enzyme-producing isolates. Out of the three strains, the two which were given the best result have been subjected to further identification.

### 3.2. Strains Identification

#### 3.2.1. Identification by Morphological and Biochemical Tests

The morphological and physiological characteristics of the isolated strains were compared with the data from Bergey's Manual of Determinative Bacteriology [17] who revealed that *Bacillus *sp. MA20 and *Bacillus *sp. MA21 are matching with those of *B. subtilis* group. In previous articles on taxonomy, species included in the *B. subtilis* group are the following*: B. velezensis*, *B. atrophaeus*, *B. mojavensis*, *B. malacitensis*, *B. axarquiensis*, *B. nematocida*, *B. vallismortis*, *B. subtilis*, and *B. amyloliquefaciens* [32–34]. 

The two strains are aerobic, motile, and Gram-positive rods. The smears of *Bacillus *sp. MA20 and *Bacillus *sp. MA21 were scanned by scanning electron microscope which indicates the bacterial size of the two *Bacillus* strains which was measured by slime view program software.

The results of biochemical tests of the *Bacillus *sp. MA20 and *Bacillus *sp. MA21 indicated that they are related to *B. subtilis*, and *B. amyloliquefaciens* which summarized in Table 1. 

### 3.3. Identification Based on Genetic Materials (DNA)

#### 3.3.1. DNA Extraction

The isolated DNA was analyzed by gel electrophoresis and the quality of the DNA for each sample has been identified for further investigation.

#### 3.3.2. Identification by 16S Ribosomal RNA (rRNA)

The amplified 16S rRNA gene from the DNA of *Bacillus *sp. MA20 and *Bacillus *sp. MA21 was determined using 2% agarose gel. The size of the amplified fragments was determined by using size standard (Gene ruler 50 bp–1031 bp DNA ladder). The PCR products were visualized under UV light and photographed using gel documentation system. Approximately 380 bp of 16S rRNA gene was amplified.

The PCR products were purified and sequenced using 16S forward primer. The sequences of *Bacillus *sp. MA20 and *Bacillus *sp. MA21 were deposited in national center for biotechnology information (NCBI GenBank) under the Accession numbers (HQ115599.1–HQ115600.1), respectively. The basic local alignment search tool (BLAST) algorithm was used to retrieve for homologous sequences in GenBank.

The *Bacillus *sp. MA20 revealed 98% identity to *Bacillus amyloliquefaciens *and *Bacillus subtilis* while *Bacillus *sp. MA21 revealed 97% identity to *Bacillus subtilis *and* Bacillus amyloliquefaciens*. Based on the morphological, biochemical, and molecular characteristics, the *Bacillus *sp. MA20 and *Bacillus *sp. MA21 were designated as *B. amyloliquefaciens* MA20 and *B. subtilis* MA21, respectively.

**Figure 2:**
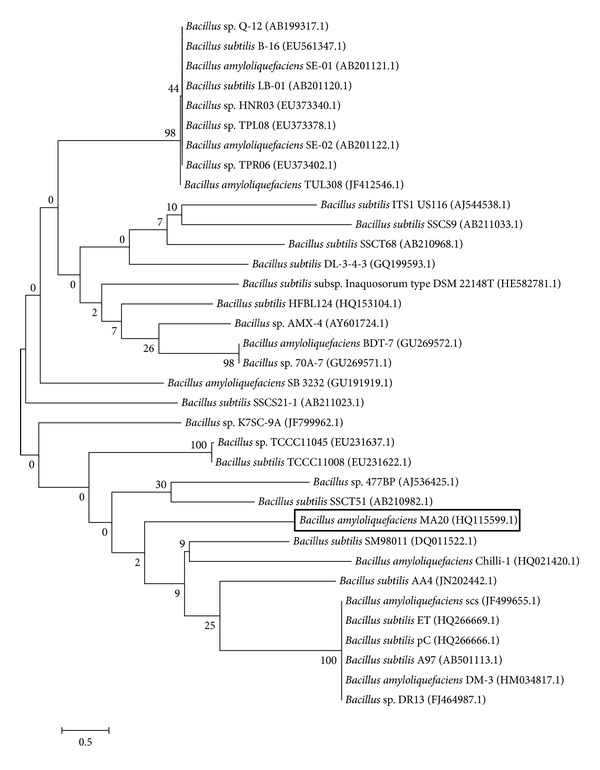
Phylogenetic position of *Bacillus amyloliquefaciens* MA20 within the genus *Bacillus*. The branching pattern was generated by neighbor-joining tree method. The Genbank accession numbers of the 16S rRNA nucleotide sequences are indicated in brackets. The number of each branch indicates the bootstrap values. The bar indicates a Jukes-Cantor distance of 0.5.

**Figure 3:**
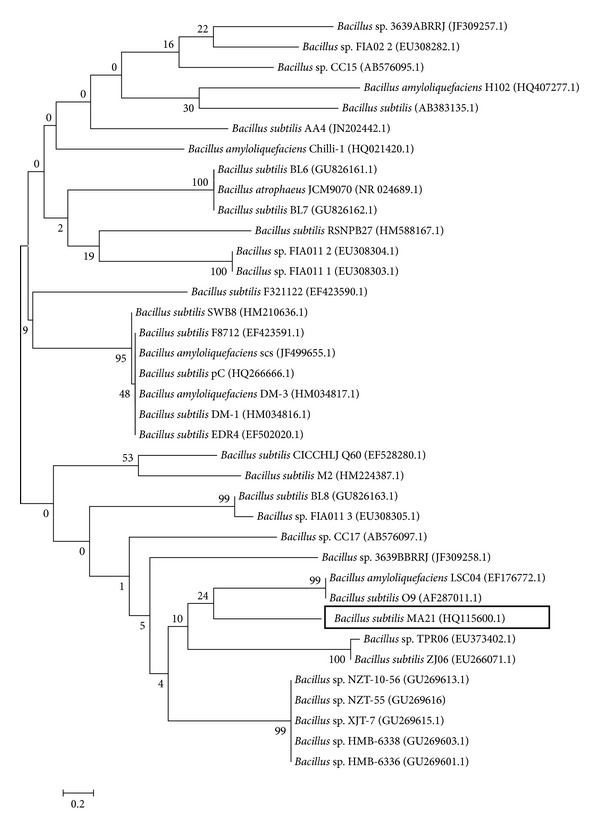
Phylogenetic position of *Bacillus subtilis* MA21 within the genus *Bacillus*. The branching pattern was generated by neighbor-joining tree method. The Genbank accession numbers of the 16S rRNA nucleotide sequences are indicated in brackets. The number of each branch indicates the bootstrap values. The bar indicates a Jukes-Cantor distance of 0.2.

A phylogenetic tree based on the comparison of 16S rRNA sequences of reference strains was constructed. The phylogenetic analysis was performed with 341 bp sequences using the software MEGA 5 [20], using the neighbour-joining method and based on Jukes-Cantor distances. The branching pattern was checked by 500 bootstrap replicates (Figures 2 and 3).

### 3.4. Media Screening for Keratinolytic Protease Production

Keratinolytic proteases are largely produced in a basal medium with keratinous substrates, and most of the organisms could utilize keratin sources such as feather and wool as the sole source of carbon and nitrogen [35, 36]. *B. amyloliquefaciens* MA20 and *B. subtilis* MA21 were tested on eight nutrient media. The selected media is medium (1) which is containing (w/v) NaCl, 0.5 g/L; K_2_HPO_4_, 0.3 g/L; KH_2_PO_4_, 0.4 g/L; wool, 10 g/L, and the pH was adjusted 7.0–7.2 using 2N of NaOH and HCl.  

Korniłłowicz-Kowalska, (1997) and Amara and Serour (2008) reported that the mass loss of the keratin substrate is the most reliable indicator of microbial keratinolytic abilities. *B. amyloliquefaciens *MA20 and B.* subtilis *MA21 have the ability to degrade wool completely after incubation for 5 days and remain it as powders in the bottom of flasks [24, 37].

### 3.5. Enzyme Production and Characterization

The keratinolytic protease enzyme was produced using production medium mentioned above for each of *B. amyloliquefaciens* MA20 and *B. subtilis* MA21, and the crude enzymes were characterized.

### 3.6. Detection of the Proteolytic Activity on Plates

**Figure 4:**
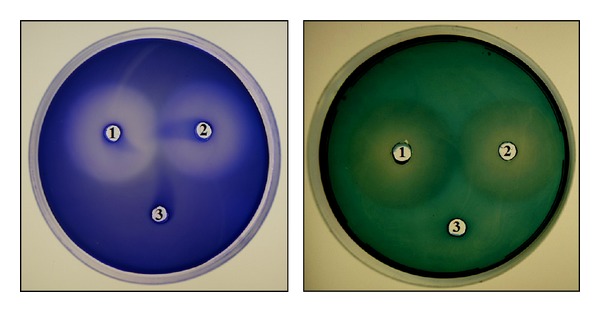
Proteolytic activity of crude enzymes produced by *B. amyloliquefaciens* MA20 and *B. subtilis* MA21 using gelatin as substrate and both of Coomassie blue and amido black stains. Blue plate stained with Coomassie blue while green plate stained with Amido black. The well (1) refers to enzymes produced by *B. amyloliquefaciens* MA20, (2) refers to enzymes produced by *B. subtilis *MA21, and (3) is inactive enzyme.

The crude enzymes of *B. amyloliquefaciens* MA20 and *B. subtilis* MA21 were assayed by agar well diffusion methods on plates containing gelatin soluble in glycine/NaOH buffer of pH 9. The Coomassie blue and amido black bind to gelatin and whole plate giving the colour of dye except the hydrolysis areas which appear as transparent without dye (Figure 4). 

### 3.7. Characterization of Keratinolytic Protease Enzyme

#### 3.7.1. Influence of pH and Temperature

The effect of temperature and pH on enzymes activity and stability was determined. The optimum temperature and pH for protease activity of enzymes produced by *B. amyloliquefaciens* MA20 and *B. subtilis* MA21 was found to be 60°C, 9.0, respectively.

**Figure 5:**
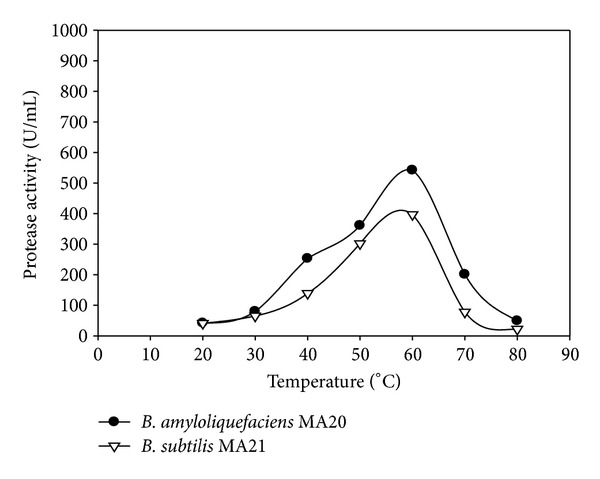
Effect of temperatures at pH 7 on protease activity of crude enzymes produced by *B. amyloliquefaciens* MA20 and *B. subtilis* MA21.

**Figure 6:**
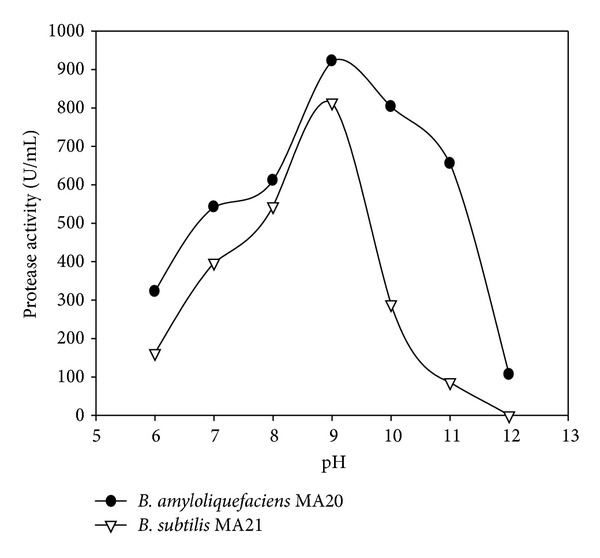
Effect of pH at 60°C on protease activity of crude enzymes produced by *B. amyloliquefaciens* MA20 and *B. subtilis* MA21.

**Figure 7:**
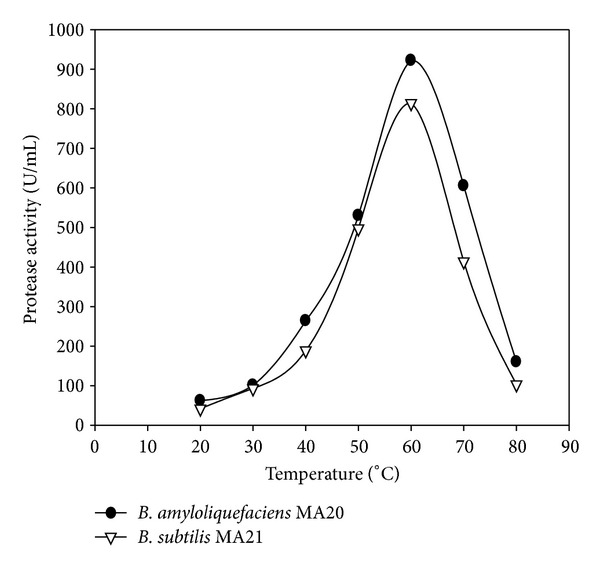
Effect of temperatures at pH 9 on protease activity of crude enzymes produced by *B. amyloliquefaciens* MA20 and *B. subtilis* MA21.

The enzymes activity was investigated at pH 7 and different temperatures (Figure 5). The proteolytic activities were determined at optimized temperature 60°C and different pH (Figure 6). For confirmation from the optimized reaction, the enzymes activity was assayed at pH 9 with different temperatures (Figure 7).

Results revealed that the keratinolytic protease from *B. amyloliquefaciens* MA20 and *B. subtilis* MA21 is similar to those produced using bacteria, actinomycetes, and fungi and has a pH optimum in a neutral-to-alkaline range [38, 39]. The optimal temperature for activity was also found in the usual range for keratinolytic protease (30–80°C). 

The maximum activity of protease enzyme produced by *B. amyloliquefaciens* MA20 was 922 U/mL at pH 9 and 60°C while the maximum activity of protease enzyme produced by *B. subtilis* MA21 was determined as 814 U/mL at the same conditions. The alkaline pH of the keratinolytic protease enzyme from *B. amyloliquefaciens* MA20 and *B. subtilis* MA21 suggests a positive biotechnological potential.

**Figure 8:**
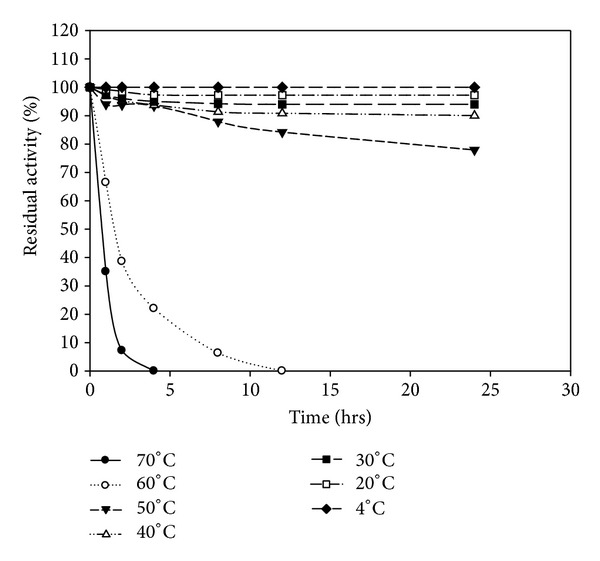
Thermal stability of crude enzyme produced by *B. amyloliquefaciens* MA20.

**Figure 9:**
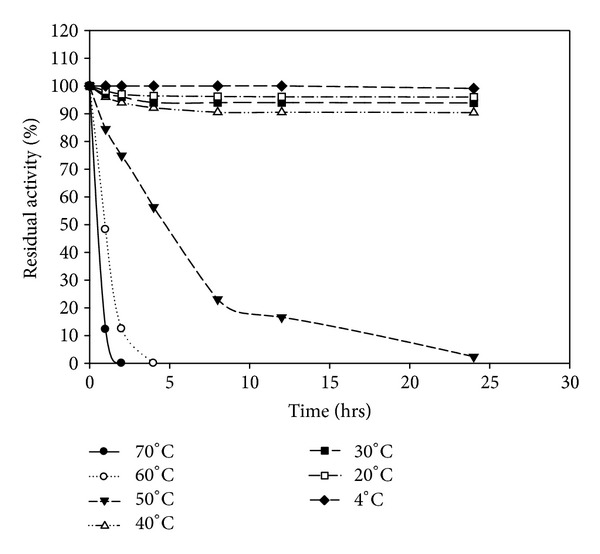
Thermal stability of crude enzyme produced by *B. subtilis* MA21.

The enzymes were stable at temperatures between 4 and 70°C. The enzyme produced by *B. amyloliquefaciens* MA20 is more thermostable than enzyme produced by *B. subtilis* MA21 (Figures 8 and 9). The results of thermal stability indicated that the keratinolytic protease from *B. amyloliquefaciens* MA20 is more thermostable than the enzyme produced by *B. subtilis* MA21.

The thermal stability studies give the enzyme from *B. amyloliquefaciens* MA20 the advantage for using in industrial applications which are the main objective of this study. 

**Figure 10:**
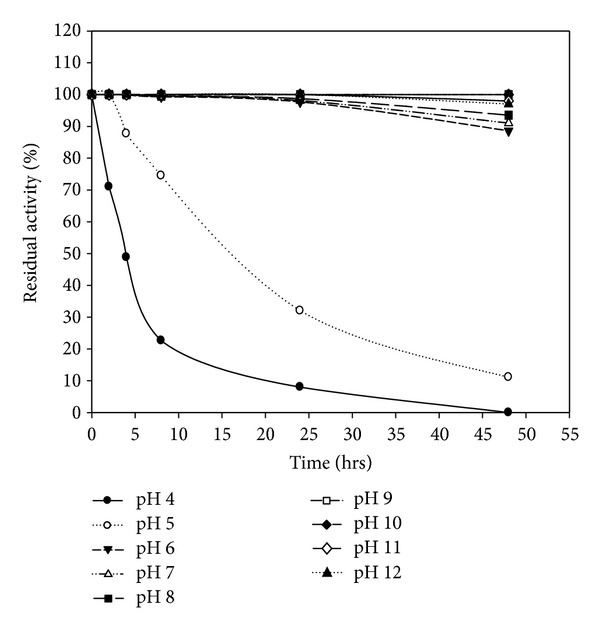
pH stability of crude enzyme produced by *B. amyloliquefaciens* MA20.

**Figure 11:**
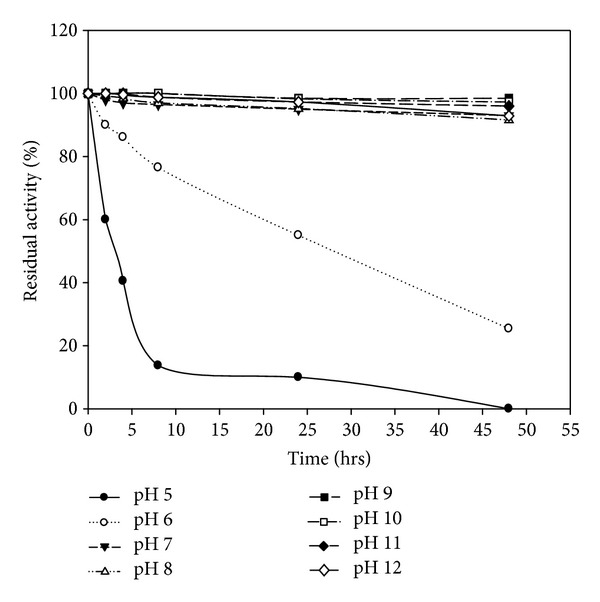
pH stability of crude enzyme produced by *B. subtilis* MA21.

The crude enzyme from *B. amyloliquefaciens* MA20 is described as stable over a broad pH range of 4.0–12.0, but the best stable pH is 9 and 10 (Figure 10).  Figure 11 indicates that the enzyme from *B. subtilis* MA21 was stable in pH range (5.0–12.0) with high stability at pH 9. The stability of keratinolytic proteases produced by *B. amyloliquefaciens* MA20 and *B. subtilis* MA21 has been suggested to offer great advantages for industrial purposes such as wastewater treatment and leather tanning [40].

### 3.8. Influence of Protease Inhibitors, Solvents, and Metal Ions on Enzymes Activity

Mostly keratinolytic proteases belong to the subtilisin family of serine proteases with cysteine proteases, which have higher activity on casein [41]. The keratinolytic proteases produced by *Bacillus *sp. are often serineproteases, such as the enzymes produced by *B. licheniformis* [42], *B. pseudofirmus* [43], and *B. subtilis* [44, 45]. Protease activity of enzymes prepared from *B. amyloliquefaciens* MA20 and *B. subtilis* MA21 was completely inhibited by serine protease inhibitor (PMSF). The result indicated the presence of the serine group in the enzyme active site. The enzymes activity was partially inhibited by EDTA (Tables 2 and 3). This suggests that the keratinolytic protease from the *Bacillus *strain belongs to keratinolytic serine protease family.

The stability of keratinolytic proteases in presence of SDS acts as a positive advantage of enzymes feature because it indicated the possibility of using them in different industrial purposes as detergent industry, leather industry, and wool improvement. The stability toward SDS is important because a few authors reported that SDS-stable enzymes are also not generally available except for a few strains such as *Bacillus clausii* I-52 [46] and *Bacillus *sp. RGR-14 [47]. The effects of various solvents and metal ions on enzyme activity were examined in order to find which ions are stimulators and which are inhibitors of the catalytic process. 

The metal ions were used with two final concentrations (5.0 mM and 10 mM) while the used solvents with final concentrations (1% and 0.5%). The effects of metal ions and solvents on enzyme activities are summarized in Tables 2 and 3.

### 3.9. First-Dimension Protein Electrophoresis

**Figure 12:**
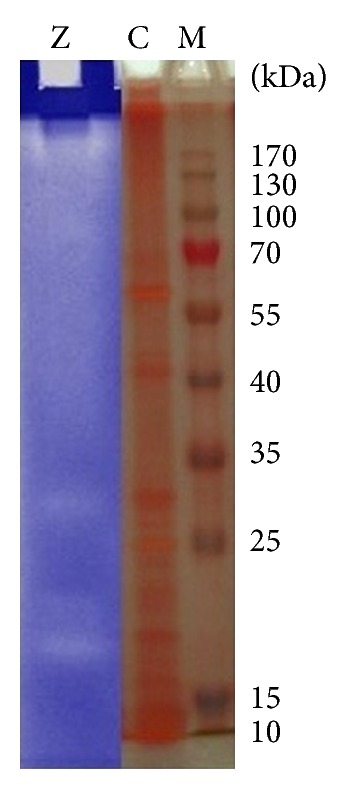
SDS-PAGE and zymogram analysis of keratinolytic protease enzyme from *B. amyloliquefaciens* MA20. The lane (M) is prestained protein ladder, lane (C) is protein pattern of crude enzyme, and lane (Z) is zymogram of enzyme.

**Figure 13:**
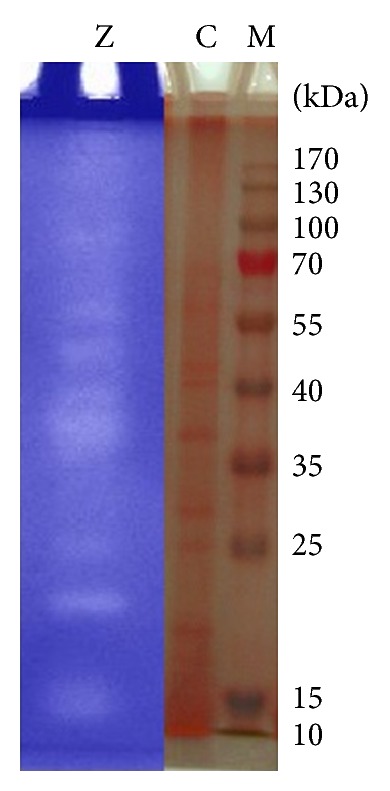
SDS-PAGE and zymogram analysis of keratinolytic protease enzyme from *B. subtilis* MA21. The lane (M) is prestained protein ladder, lane (C) is protein pattern of crude enzyme, and lane (Z) is zymogram of enzyme.

**Figure 14:**
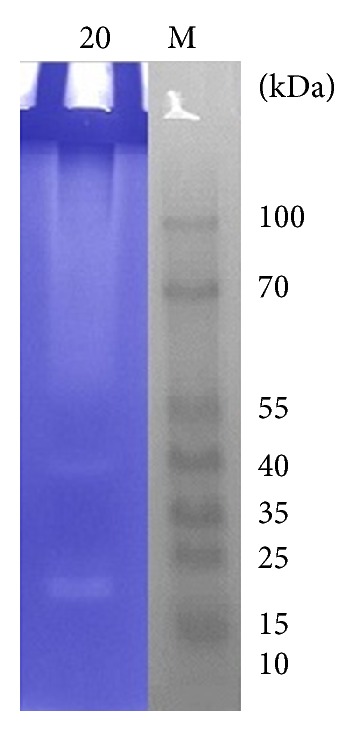
Native-PAGE zymogram analysis of keratinolytic protease enzyme from *B. amyloliquefaciens* MA20. The lane (M) is prestained protein ladder, and lane (20) is zymogram of enzyme.

**Figure 15:**
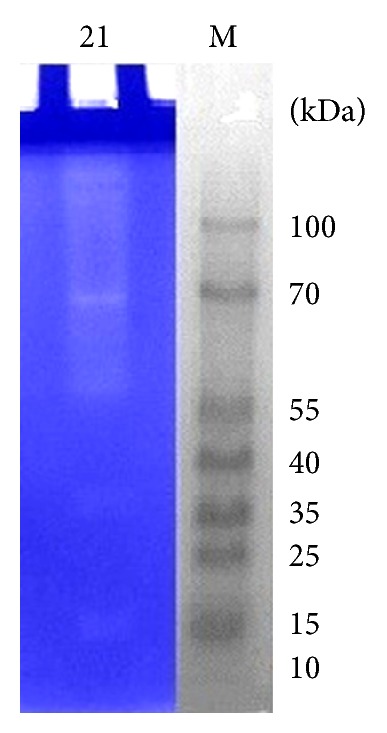
Native-PAGE zymogram analysis of keratinolytic protease enzyme from *B. subtilis* MA21. The lane (M) is prestained protein ladder, and lane (21) is zymogram of enzyme.

The production of extracellular keratinolytic proteases by *B. amyloliquefaciens* MA20 and *B. subtilis* MA21 was evaluated by SDS-PAGE and zymogram analysis. Giongo et al. found multiple bands after zymogram of keratinolytic protease produced by three strains of *Bacillus sp*. P6, P7, and P11 using feather degrading medium [25]. Growth of the two strains in presence 10 g/L of wool resulted in the production of multiple proteases, as observed by a zymogram on gelatin (Figures 12 and 13). Multiple clear zones were observed and detected using (PAGE Ruler Prestained Protein Ladder) which indicated that the proteolytic activity was not due to a single protein. The native-PAGE zymogram of enzymes from the 2 strains was performed and compared with protein ladder (Figures 14 and 15). The zymogram using native page was carried out for detecting the bands of protein where the purification performs using native protein. Multiple bands could be detected on native-PAGE zymogrm.

### 3.10. Two-Dimension Protein Electrophoresis

The two-dimensional polyacrylamide gel electrophoresis (2D-PAGE) is an advanced technique that depends on protein separation firstly according to the pH and secondly based on the molecular weight. This technique was carried out to differentiate between the crude keratinolytic proteases obtained from *B. amyloliquefaciens* MA20 and *B. subtilis* MA21. After the separation based on the pH, the strips were applied on gel for separating according to molecular weight. The gels were stained with Coomassie blue for detecting the protein spots.

**Figure 16:**
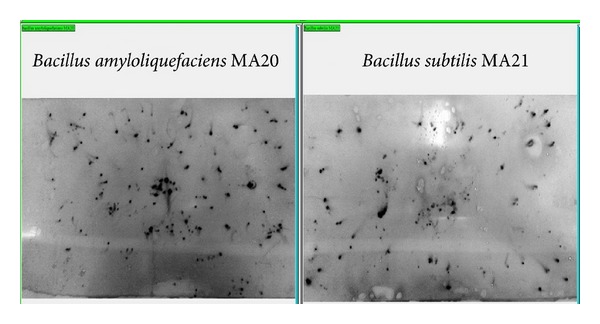
Two-dimension gel of enzymes from *B. amyloliquefaciens* MA20 and *B. subtilis* MA21.

In order to classify the keratinolytic protease enzyme, a systemic comparison of 2D maps of proteases was conducted with image master 2D Platinum 6 software. The two gels were photographed by high quality scanner image (Figure 16). 

The results were analyzed using image master 2D Platinum 6 software. The spots were detected before matching between the two gels and the report was obtained (Table 4). Every spot refers to the presence of one protein. The gel which was loaded with keratinolytic protease from *B. amyloliquefaciens* MA20 had 237 spots while the gel applied with *B. subtilis* MA21 had 291 spots.

The 2 gels were matched and consider gel resulted from crude enzymes of *B. amyloliquefaciens* MA20 as reference and the final report that summarizes the difference between 2 gels was obtained (Table 5). The 2 gels were matched and the percent of matching between them was 13.25%.

## 4. Conclusion

This paper described in details different methods that lead to the production of keratinolytic protease from two *Bacillus *sp. strains. Different methods and assays ranging from simple to advanced were carried out to prove that the enzymes have the ability to degrade wool. The two used strains have been selected based on that they belong to *Bacillus *sp. and show the best keratinolytic activities. The study included molecular and bioinformatic tools to identify the two *Bacillus *sp. strains. 16S rRNA, phylogenetic tree, Blast search for nucleotide similarity, scanning electron microscope, SDS-PAGE, and 2D-PAGE have been used to differentiate the both strains and their produced enzymes. The study succeeded in characterization of *Bacillus *sp. strains which were named *Bacillus amyloliquefaciens* MA20 and *Bacillus subtilis* MA21. The enzyme activities either on agar diffusion plates or through the enzyme activity bioassays under different experimental conditions proved that the both strains are able to produce different keratinolytic protease enzymes. *B. amyloliquefaciens* MA20 and *B. subtilis* MA21 could be used in large-scale production of keratinolytic protease enzymes where these enzymes were stable at temperature range between 4 and 70°C, and over a wide range of pH values (4–12), as well as, stable against organic solvents and detergents. The characters of keratinolytic enzymes produced by *B. amyloliquefaciens* MA20 and *B. subtilis* MA21 could play an important role especially in industrial applications; therefore this research acts as preliminary studies for applying the keratinolytic proteases in wool quality improvement.

## Supplementary Material

Scanning electron microscope of Bacillus amyloliquefaciens MA20 and Bacillus subtilis MA21
The supplementary data include the characterization of Bacillus amyloliquefaciens MA20 and Bacillus subtilis MA21 using scanning electron microscope as in figure 1 which indicates to the bacterial size of the 2 Bacillus strains which measured by slime view program software.
DNA isolation of Bacillus amyloliquefaciens MA20 and Bacillus subtilis MA21
The genomic DNA of B. amyloliquefaciens MA20 and B. subtilis MA21 were isolated and purified. The DNA was analyzed by gel electrophoresis using 1% (w/v) agarose gel containing ethidium bromide soluble in TBE buffer were used. The DNA ladder was loaded in gel for detecting the DNA. The DNA was investigated under UV light using gel documentation system and photographed as in figure 2.
16S ribosomal RNA (rRNA)
The amplified 16S rRNA gene from the DNA of B. amyloliquefaciens MA20 and B. subtilis MA21 were determined using 2% agarose gel. The PCR products were about 380 bp in compare to DNA ladder (Gene ruler 50 bp – 1031 bp DNA ladder) (Figure 3).
Production of keratinolytic proteases
The keratinolytic proteases were produced from B. amyloliquefaciens MA20 and B. subtilis MA21 using medium which containing wool as sole carbon and nitrogen source. The wool was degraded to powder after incubation for 5 days (figure 4).

## Figures and Tables

**Table 1:** Morphological and biochemical properties of *Bacillus *sp. MA20 and *Bacillus *sp. MA21.

Characteristics	*Bacillus *sp. MA20	*Bacillus *sp. MA21
Morphological		
Shape	Rods	Rods
Gram stain	G+ve	G+ve
Motility	Motile	Motile
spore formation	+ve	+ve
Growth		
Growth temperature	15°C–50°C	15°C–60°C
Growth pH	5–8	5–8
Biochemical tests		
Oxidase	+ve	+ve
Catalase	+ve	+ve
Voges-Proskauer	+ve	+ve
Indol production	−ve	−ve
Nitrate reduced to nitrite	+ve	+ve
Hydrolysis of		
Casein	+ve	+ve
Gelatin	+ve	+ve
Wool	+ve	+ve
Starch	+ve	+ve
Acid from		
Glucose	+ve	+ve
Arabinose	+ve	+ve
Xylose	+ve	+ve
Mannitol	+ve	+ve
Gas from glucose	−ve	−ve
Utilization of		
Citrate	+ve	+ve
Propionate	−ve	−ve

**Table 2:** Effect of protease inhibitors, metal ions, and solvents on proteolytic activity of *B. amyloliquefaciens* MA20.

Substance	Final concentration	Relative activity (%)	Final concentration	Relative activity (%)
Control	0	100	0	100
**β**-mercaptoethanol	5 mM	18	1 mM	98
PMSF	5 mM	0	1 mM	0
EDTA	5 mM	35	1 mM	97
SDS	0.5%	54	0.1%	122
ZnCl_2_	10 mM	153	5 mM	156
MgCl_2_	10 mM	119	5 mM	95
CuSO_4_	10 mM	266	5 mM	204
Urea	10 mM	114	5 mM	139
HgCl_2_	10 mM	100.4	5 mM	107
CaCl_2_	10 mM	104	5 mM	107
BaCl_2_	10 mM	115	5 mM	116
Guanidin HCl	10 mM	103	5 mM	132
MnCl_2_	10 mM	71	5 mM	162
Methanol	1%	90	0.5%	100
Ethanol	1%	103	0.5%	100
DMSO	1%	90	0.5%	103
Isopropanol	1%	98	0.5%	108
Tween 20	1%	86	0.5%	94
Triton X100	1%	61	0.5%	65

**Table 3:** Effect of protease inhibitors, metal ions, and solvents on proteolytic activity of *B. subtilis* MA21.

Substance	Final concentration	Relative activity (%)	Final concentration	Relative activity (%)
Control	0	**100**	0	100
*β*-mercaptoethanol	5 mM	48	1 mM	70
PMSF	5 mM	0	1 mM	0
EDTA	5 mM	13	1 mM	96
SDS	0.5%	25	0.1%	99
ZnCl_2_	10 mM	**92**	5 mM	93
MgCl_2_	10 mM	**22**	5 mM	98
CuSO_4_	10 mM	**23**	5 mM	73
Urea	10 mM	**24**	5 mM	52
HgCl_2_	10 mM	**27**	5 mM	37
CaCl_2_	10 mM	**65**	5 mM	142
BaCl_2_	10 mM	**29**	5 mM	69
Guanidin HCl	10 mM	**42**	5 mM	49
MnCl_2_	10 mM	**64**	5 mM	67
Methanol	1%	92	0.5%	99
Ethanol	1%	92	0.5%	97
DMSO	1%	78	0.5%	96
Isopropanol	1%	98	0.5%	101
Tween 20	1%	78	0.5%	86
Triton X100	1%	57	0.5%	133

**Table 4:** Two-dimension protein gel electrophoresis report of crude enzymes produced by *B. amyloliquefaciens *MA20 and *B. subtilis *MA21.

Gels	Spots	Minimum gray	Maximum gray	Columns	Rows	Pixel width	Pixel height
*B. amyloliquefaciens* MA20	237	23	209	2512	1510	353	353
*B. subtilis *MA21	291	0	255	2336	1452	353	353

**Table 5:** Match statistics report of crude enzymes produced by *B. amyloliquefaciens *MA20 and *B. subtilis *MA21 after two-dimension protein gel electrophoresis.

Gel name	Gel name	Number of matches	Percent matches
*B. amyloliquefaciens* MA20 (reference)	*B. subtilis *MA21	35	13.2576
