# Supplementary material for: Production and Characterization of Keratinolytic Protease from New Wool-Degrading Bacillus Species Isolated from Egyptian Ecosystem
Source: Biomed Res Int. 2013 Jul 11;2013:175012. doi: 10.1155/2013/175012 (PMC3725791; doi:10.1155/2013/175012)
Supplement: Supplementary file 1 — Scanning electron microscope of Bacillus amyloliquefaciens MA20 and Bacillus subtilis MA21 The supplementary data include the characterization of Bacillus amyloliquefaciens MA20 and Bacillus subtilis MA21 using scanning electron microscope as in figure 1 which indicates to the bacterial size of the 2 Bacillus strains which measured by slime view program software. DNA isolation of Bacillus amyloliquefaciens MA20 and Bacillus subtilis MA21 The genomic DNA of B. amyloliquefaciens MA20 and B. subtilis MA21 were isolated and purified. The DNA was analyzed by gel electrophoresis using 1% (w/v) agarose gel containing ethidium bromide soluble in TBE buffer were used. The DNA ladder was loaded in gel for detecting the DNA. The DNA was investigated under UV light using gel documentation system and photographed as in figure 2. 16S ribosomal RNA (rRNA) The amplified 16S rRNA gene from the DNA of B. amyloliquefaciens MA20 and B. subtilis MA21 were determined using 2% agarose gel. The PCR products were about 380 bp in compare to DNA ladder (Gene ruler 50 bp – 1031 bp DNA ladder) (Figure 3). Production of keratinolytic proteases The keratinolytic proteases were produced from B. amyloliquefaciens MA20 and B. subtilis MA21 using medium which containing wool as sole carbon and nitrogen source. The wool was degraded to powder after incubation for 5 days (figure 4). [file 175012.f1.pdf]

Supplementary data

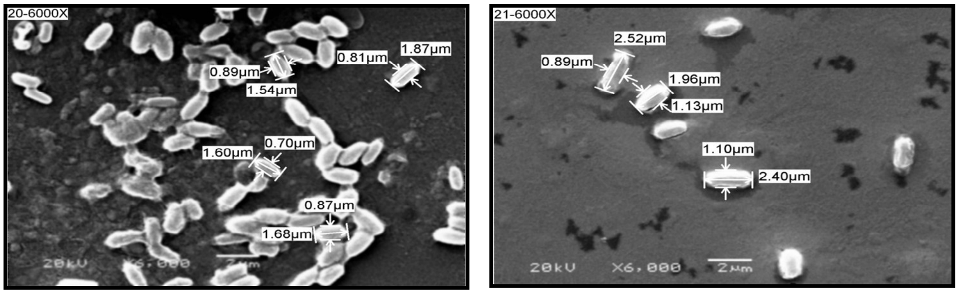

(A) **(B)**

Figure 1: Scanning electron microscope of *Bacillus sp.* MA20 and *Bacillus sp.* MA21 at 6000X with bacterial size. (Fig. A) is *Bacillus sp.* MA20 while (fig. B) is *Bacillus sp.* MA21.

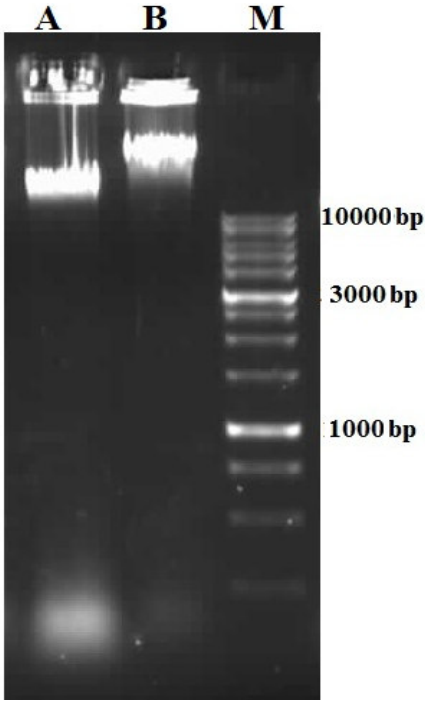

Figure 2: DNA of *Bacillus sp.* MA20 and *Bacillus sp.* MA21 separated in 1% agarose gel. The lane (A) is DNA from *Bacillus sp.* MA20, lane (B) is DNA from *Bacillus sp.* MA21, and lane (M) is 1 Kb DNA ladder.

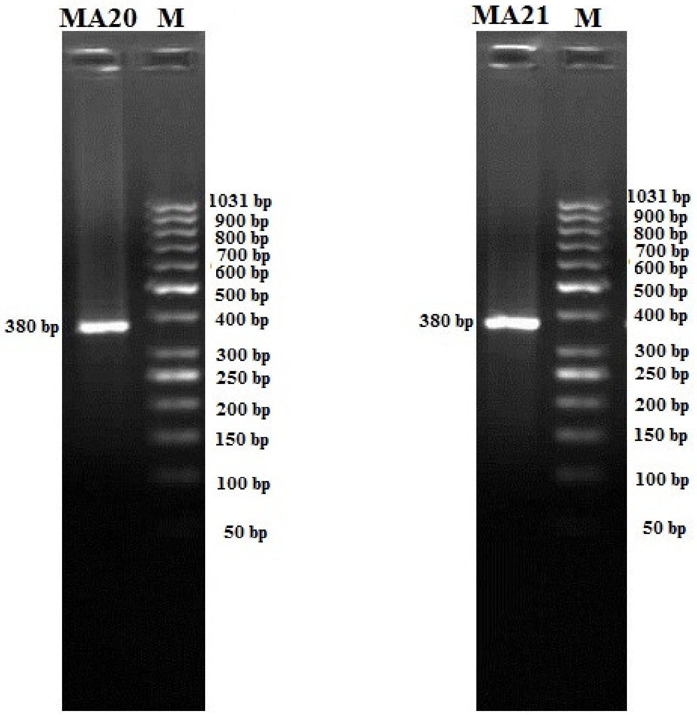

(A) **(B)**

Figure 3: Amplified 16S rRNA gene from the DNA of *Bacillus sp.* MA20 and *Bacillus sp.* MA21 on 2% agarose gel. Lane (MA20) is from *Bacillus sp.* MA20, lane (MA21) is from *Bacillus sp.* MA21 and lane (M) is DNA ladder.

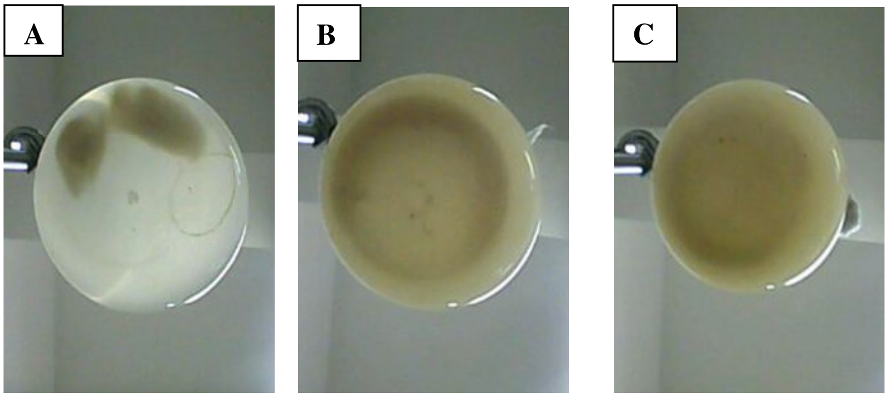

Figure 4: Flask containing wool degraded in keratinolytic protease production medium by *B. amyloliquefaciens* MA20 and *B. subtilis* MA21. Figure (A) is control medium, figure (B) is wool degraded by *B. amyloliquefaciens* MA20, and figure (C) is wool degraded by *B. subtilis* MA21.
